# Comprehensive Profiling of Illicit Amphetamines Seized in Poland: Insights from Gas Chromatography–Mass Spectrometry and Chemometric Analysis

**DOI:** 10.3390/molecules30030579

**Published:** 2025-01-27

**Authors:** Anna Czyż, Katarzyna Pawlak, Emilia Waraksa, Tomasz Bieńkowski

**Affiliations:** 1 Faculty of Chemistry, Warsaw University of Technology, Noakowskiego 3, 00-664 Warsaw, Poland; 2 Masdiag DNA Centrum Ekspertyz Kryminalistycznych, Żeromskiego 33, 01-882 Warsaw, Poland; 3 Masdiag, Żeromskiego 33, 01-882 Warsaw, Poland; tomasz.bienkowski@masdiag.pl

**Keywords:** amphetamine, chemical impurity profiling, chemometric techniques, gas chromatography with mass spectrometry

## Abstract

The illicit production and distribution of amphetamines present significant challenges to public health and law enforcement, particularly in Europe, where these substances dominate the stimulant market. This study aimed to profile amphetamines consumed within a Polish community by employing gas chromatography–mass spectrometry (GC-MS) and chemometric techniques to analyze their chemical composition and associated impurities. The optimized GC-MS methodology facilitated the identification of synthesis markers, precursor origins, and distribution patterns. Impurity profiling provided critical insights into regional production trends, including the use of specific precursors and adulterants. Chemometric analysis further enabled the classification of samples into distinct groups, shedding light on their origins and distribution chains. These findings underscore the potential of extending amphetamine profiling to include distribution-related compounds, offering a powerful tool for tracking production trends and enhancing forensic investigations in the fight against drug trafficking.

## 1. Introduction

Millions worldwide are affected by the use of illegal substances like cocaine, opioids, psychedelics, and amphetamines, posing serious public health, social, and legal challenges [1]. Drug trafficking and use demand stricter law enforcement and regulations to address these issues [2]. Despite disruptions from the SARS-CoV-2 pandemic and armed conflicts, illicit drug manufacturers continue to adapt by altering product potency, using novel materials, and refining distribution. Europe remains a key hub for amphetamine production [3], with a market worth over €1.1 billion annually and numerous synthetic drug facilities [4].

The largest amphetamine production hub is in northwestern Europe, primarily in the Netherlands and Belgium, where amphetamine and ecstasy tablets are primarily manufactured [5,6]. Shipments of amphetamine freebase from the Netherlands are converted to amphetamine sulfate in other countries [7]. Northeastern Europe, including Poland, the Czech Republic, Lithuania, and Estonia, forms the second-largest hub. The Czech Republic is known for small-scale methamphetamine (“Pervitin”) production in home labs using ephedrine or pseudoephedrine [8,9]. Poland produces amphetamine derivatives and cathinones, mainly as powder or paste [10]. Southeastern Europe, including Bulgaria, Serbia, and Turkey, focuses on amphetamine tablets, often as counterfeit “Captagon,” containing amphetamine and caffeine instead of phenethylline [11,12].

In Europe, including Poland, amphetamine production is largely dominated by organized crime groups operating advanced laboratories [13,14]. These facilities feature professional-grade steel reactors, vacuum distillation systems, and high-capacity setups capable of producing up to 40 kg per cycle, a fivefold increase in efficiency without compromising quality. This shift away from traditional glassware setups reflects significant technological advancements [15,16]. Despite a decline in the number of production sites, the growing capacity of existing labs significantly hampers efforts to track and interpret amphetamine production dynamics [17].

The synthesis of amphetamine can be achieved through numerous methods, with one of the most commonly used approaches being the reductive amination of benzyl methyl ketone (BMK, also known as 1-phenyl-2-propanone, P2P). The Leuckart reaction is primarily employed in illicit amphetamine manufacturing, effectively converting ketones into amines [18], alongside other techniques like nitrostyrene reaction [19]. Seldom, and on a limited basis, efforts are made to discover entirely novel synthesis methods. As a result of tighter controls on precursor chemicals, criminal groups are being forced to employ more complex production methods. This process typically involves using different starting materials that can be converted into the desired precursors.

BMK is commonly produced in Russia and China [17]. However, increased international cooperation has made obtaining BMK significantly more challenging. This is reflected in the presence of amphetamines contaminated with 1-phenylethylamine (1-PEA) on the drug market. To boost production volumes, illicit manufacturers often add acetophenone, which undergoes similar chemical transformations, leading to the formation of 1-PEA as a byproduct [20]. As restrictions tightened, producers began turning to unregulated chemicals that can be readily converted into BMK, bypassing existing controls [21]. In the early 2000s, phenylacetic acid (PAA) became a widely used pre-precursor [22]. In 2008, the Netherlands reported the emergence of a masked precursor, known as the BMK hydrosulfite adduct [23]. By 2010, alpha-phenylacetoacetonitrile (APAAN) had entered the market [7,24], and in 2013, 3-oxo-N-phenylbutanamide was identified as a “pre-pre-precursor”, a compound readily convertible into APAAN. [25]. Consequently, APAAN was placed under international control by late 2014, prompting manufacturers to seek alternative precursors. These included alpha-phenylacetoacetamide (APAA) [26], glycidic acid derivatives in 2015, methyl 3-oxo-2-phenylbutanoate (MAPA) by late 2017, and ethyl 3-oxo-2-phenylbutanoate (EAPA) in 2021 [27]. In 2022, a number of new substances emerged, including diethyl(phenylacetyl)propanedioate (DEPAPD) [4] and three others: 3-oxo-4-phenyl-butyric acid ethyl ester, 3-oxo-2-phenylbutanoic acid, and methyl-3-oxo-2-phenylbutyrate [28]. Additionally, various salts (such as sodium (Na^+^) and potassium (K^+^)) and esters of BMK methyl glycidic acid were reported for the first time [23]. At present, 1-phenyl-2-nitropropene is one of the frequently used alternatives [29].

The final step of the synthesis process involves transforming the amphetamine into amphetamine sulfate, resulting in a powder that is white or off-white in color. To increase profitability before being sold on the street, the substance is often mixed with various chemicals, primarily aspirin, creatine, and caffeine [16]. At the final stages of the distribution chain, the content of the pure drug is lowered to as little as several percent [10].

The product available on the market consists of various chemical substances linked to its manufacturing process (substrates, reagents, intermediate products, and byproducts of side reactions), along with various additives and diluents. A chromatographic analysis of amphetamine seizures yields a chemical fingerprint that offers detailed information for characterizing, comparing, and distinguishing between samples. The profiling process can identify the origin and synthesis method used to produce the drug. The UN General Assembly Plenary on 10 June 1998 adopted the ‘Plan of action against the illicit production, trafficking and abuse of amphetamine-type stimulants and their precursors’, which suggested, among other things, profiling of these substances [30].

The profiling concept stems from the research conducted by Lars Strömberg, who employed gas chromatography (GC) with FID and ECD detection to analyze the impurities of amphetamine [31], phenmetrazine [32], and methamphetamine [33]. So far, profiling analysis has been conducted with GC equipped with FID detectors [34,35,36,37,38,39,40,41,42,43], ECD [44], NPD, MS [23,35,45,46,47,48,49,50,51,52,53,54,55,56,57,58,59,60,61], and MS/MS [62] analyzers. Additionally, TLC has been utilized for the swift identification of contaminants [63]. The analytical methods used in chemical drug profiling besides those already mentioned comprise NMR [64,65,66], HPLC with UV [67,68,69], PDA [70], DAD [71,72,73,74], MS [75], MS/MS [76,77,78,79,80,81], TOF [82,83] detection, capillary electrochromatography coupled with LIF detection [70,84], capillary electrophoresis with UV [85,86], DAD [87], FL [88], and MS [89] detection, as well as trace element analysis techniques such as ICP-MS [61,90,91,92,93], ICP-OES [94], and AAS [94,95]. The commercial availability of GC-MS systems has made this technique the preferred method for profiling amphetamine samples. GC-MS has a greater ability to overcome the issue of coelution among compounds with complex structures, thereby providing sufficient selectivity and sensitivity for detecting very small amounts of substances within intricate mixtures. GC-MS-based analyses can be more cost-effective and have a lower environmental impact than LC-MS. A research project supported by the European Union between 2005 and 2007 produced several studies on the optimization of a standardized method for amphetamine profiling via GC-MS and chemometric techniques [96,97,98,99,100,101].

Given the evolving Polish drug market and the consequent adjustments to supply chains, the method of amphetamine profiling will require a corresponding adaptation. Large amounts of liquid amphetamine brought into Poland from the Netherlands are being converted into amphetamine sulfate, watered down, and sold. Including various diluents added during the distribution process in the profiling approach could offer significant additional insights into the analyzed samples. This new article outlines the development of a GC-MS technique for creating detailed profiles of impurities in amphetamine, including the use of chemometric approaches for classifying samples.

## 2. Results and Discussion

The identification of amphetamine in powders seized by drug enforcement agencies is enabled by gas chromatography in conjunction with mass spectrometry. This technique also enables the collection of detailed information about the composition of impurities and additives present in amphetamine-containing products when the powder’s weight surpasses 0.03 g. In this study, amphetamine and its impurities were extracted using a mixture of methanol with water (80:20), and the resulting mixture was then employed for analysis.

### 2.1. Optimization of Chromatographic Method

The GC-MS method for blood analysis was adapted from a laboratory-established technique used for detecting a broad spectrum of substances in standard forensic tests, including amphetamines, cocaine, cathinones, synthetic cannabinoids, and opiates. A mixture of the amphetamine standard at 100 μg/mL and the internal standard N,N-dimethylphenylamine (ISTD) at 25 μg/mL in methanol was used; this mixture comprised substances derived from products containing amphetamine, which were synthesized and included diluting additives, to optimize the chromatographic separation conditions. A non-polar HP-1 dimethylpolysiloxane column was employed, offering excellent thermal and chemical inertness and selectivity for a wide range of impurities, including alcohols, sulfur-containing compounds, and chlorinated aromatic compounds, alongside the expected amines. The impact of the injector’s split stream ratio, helium carrier gas flow rate, and temperature on chromatographic resolution was also evaluated, to optimize the separation process’s efficiency.

**Figure 1:**
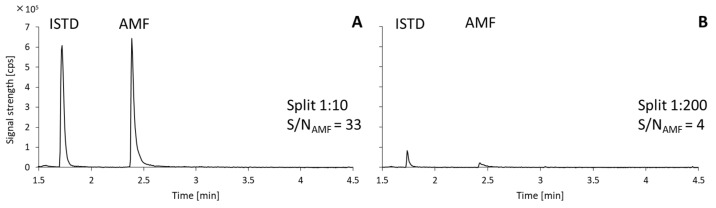
Chromatograms obtained using a flow split ratio of 1:10 (**A**) and 1:200 (**B**).

The sharpest chromatographic peaks resulted from a 1:10 sample stream split. The 1:200 split ratio of the stream transporting sample vapors from the injector was too high, resulting in the excessive dilution of amphetamine’s vapors by the carrier gas and a considerable decrease in the sensitivity of the analytical method. The signal-to-noise ratio dropped to below 10 for samples with trace amounts of analytes, rendering the detection of trace components unfeasible (Figure 1).

**Figure 2:**
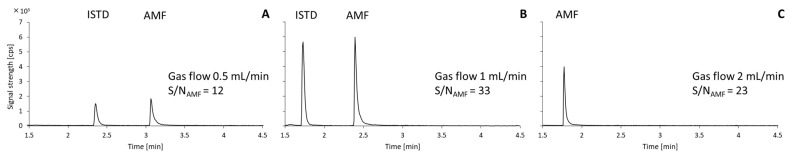
Chromatograms obtained using carrier gas flows of 0.5 mL/min (**A**), 1 mL/min (**B**), and 2 mL/min (**C**).

A more pronounced reduction in signal intensity was observed for the less volatile amphetamine compared to the internal standard (ISTD), with the amphetamine signal decreasing as the carrier gas flow increased (Figure 2). This effect is likely due to excessively low temperatures in the dispenser chamber, causing condensation of the compound. To address this, the dispenser temperature was set to 250 °C to prevent such issues. The highest chromatographic peaks were achieved at a carrier gas flow rate of 1 mL/min. At a reduced flow rate of 0.5 mL/min, the resulting peaks were lower and had longer retention times compared to those at 1 mL/min. This effect may be attributed to reduced mass exchange rates between the stationary and mobile phases and a corresponding decrease in process efficiency, as illustrated in Figure 2A, ultimately leading to a loss of sensitivity.

**Figure 3:**
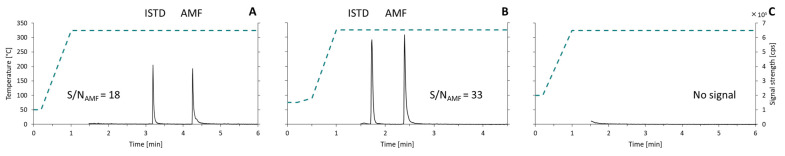
Chromatograms obtained with a gradient of 0.0–0.2 min–50 °C (0 °C/min), 0.2–1.0 min–325 °C (8 °C/min) (**A**), 0.0–0.2 min–75 °C (0 °C/min), 0.2–0.5 min–90 °C (10 °C/min), 0.5–1.0 min–325 °C (25 °C/min) (**B**), and a gradient of 0.0–0.2 min–100 °C (0 °C/min), 0.2–1.0 min–325 °C (10 °C/min) (**C**). Changes in temperature are indicated by blue dashed line.

The vapors produced from the resulting compound in the injector showed minimal variation in boiling point, yet it was essential to isolate them from the methanol. The compounds were concentrated at the head of the column and then quickly removed through evaporation. A temperature of 50 °C was found to be insufficient, resulting in a substantial decrease in mass transport efficiency between the stationary and gas phases. Performing the enrichment of the compounds at a temperature slightly below methanol’s boiling point (78.4 °C) yielded a superior outcome, which was then achieved by evaporating the methanol at 100 °C, followed by separation of the remaining compounds through an isocratic process (Figure 3). The optimal operating parameters are presented in Table 1.

### 2.2. Confirmation of Identity for Amphetamine and Qualitative Analysis for Other Compounds

**Figure 4:**
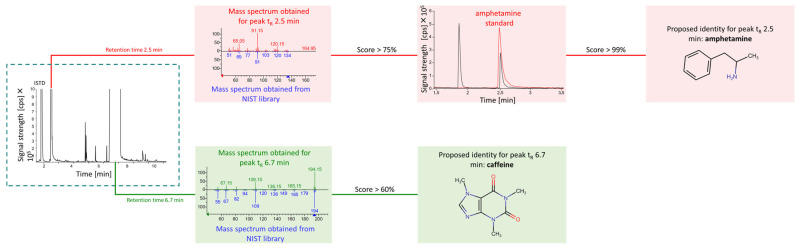
Identification of the primary constituents of a sample. The score signifies the relative similarity between experimental mass spectra and standard reference spectra.

The Enhanced Data Analysis ChemStation software (D.03.00.611, Agilent Technologies, Santa Clara, CA, USA) was employed for qualitative analysis. Identification of compounds regulated under the Anti-Drug Act was conducted by comparing the retention times obtained for samples with those of reference substances, and matching their mass spectra with those available in the National Institute of Standards and Technology (NIST) spectra library (version 17), supplemented by the SWG-DRUG database (version 3.3C) and Cayman Chemical database (version 061112), as illustrated in Figure 4. A spectral match factor exceeding 75% was required to confirm the identity of a substance. In the case of diluents and drug synthesis markers, due to limited access to such a large number of standards, identification was only carried out by comparing the mass spectrum with spectra from an expanded spectra library. The identity of the substance was considered as confirmed when the spectral match factor was above 60%.

### 2.3. Determination of Amphetamine in Obtained Samples

The concentration of amphetamine in each sample was calculated using the calibration curve equation (slope and intercept coefficients) and the observed ratio of analyte peak area to internal standard peak area (PAMF/PISTD).

The final result of the analysis was expressed as the percentage of amphetamine in the analyzed powder. Using the net mass of the secured powder and the determined amphetamine content, the following parameters were calculated [10]:

- The number of intoxicating doses, based on a standard dose of 10 mg of pure amphetamine;
- The number of doses for “heavy users”, defined as 50 mg of pure amphetamine per dose;
- The mass of the smallest intoxicating dose;
- The number of commercial portions, assumed to be 1 g per portion;
- The market value, calculated at 10 EUR per gram.

**Figure 5:**
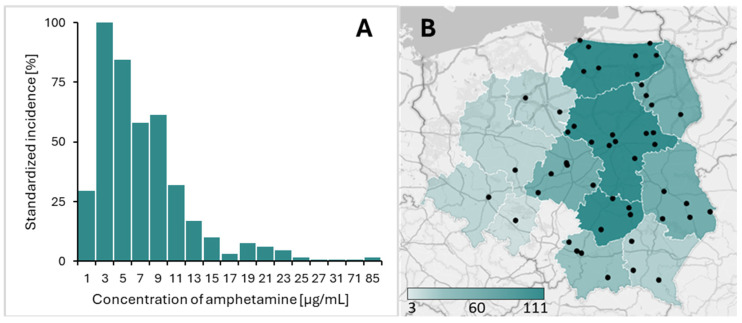
A histogram of amphetamine concentration in the 583 drug seizure samples analyzed (**A**). A map of Poland presenting the locations of the Prosecutor’s Offices and Police Stations that provided material for the investigation. The number of amphetamine samples received from each voivodeship ranged from 3 to 111 (**B**).

A total of 1744 powder evidence samples were analyzed. Amphetamine was detected in 711 samples, of which 583 samples underwent quantitative analysis. Quantitative assays were not performed on 128 samples due to insufficient sample amounts (<0.03 g). General information regarding the analyzed amphetamine samples is summarized in Table 2 and Figure 5.

Samples with confirmed and determined amounts of amphetamine were selected for further investigation, which was profiling of their impurities.

### 2.4. Data Preparation for Impurity Profiling

The samples under investigation were sorted into 48 Prosecutor’s Offices and Police Stations handling individual criminal cases (Figure 5B).

The first step in developing the profiling method was to create a database of chemical compound impurities that served as markers for those found in the actual samples. Two samples were randomly selected from each of the 48 groups and subjected to chromatographic analysis in the scanning mode, followed by a thorough qualitative analysis using the Enhanced Data Analysis ChemStation program.

**Figure 6:**
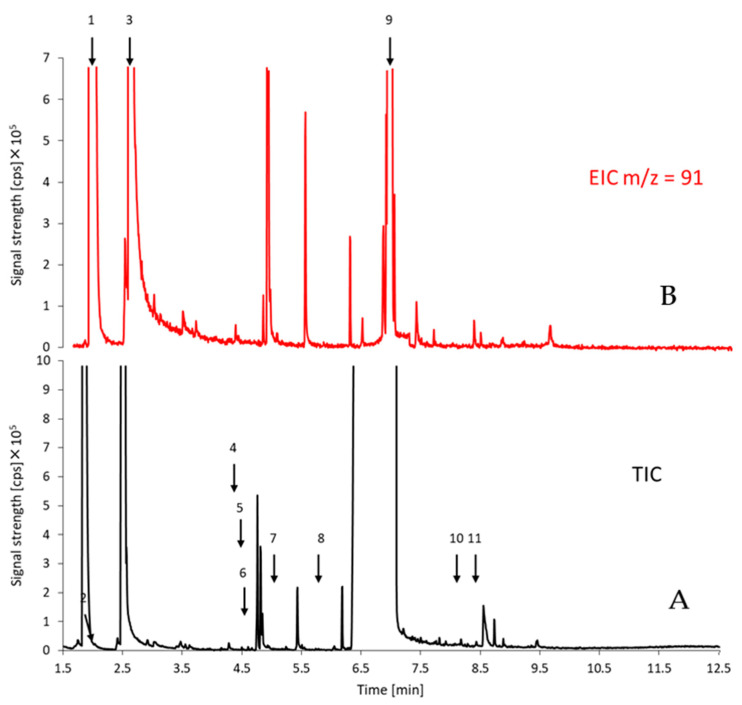
An example of the chromatograms of confiscated amphetamine powders. The identity of the compounds corresponds to the numbering in Table 3 (**A**), an extracted-ion chromatogram for the ion *m*/*z* = 91 (**B**).

In the next step, total ion chromatograms (Figure 6) were thoroughly analyzed by searching for chromatographic peaks with a height three times greater than the noise amplitude of the chromatographic baseline. The mass spectrum was then extracted for the peak and compared with the spectra in the database. When the coefficient of agreement between the spectrum of a given compound and the spectrum from the NIST database was at least 60%, the name of the compound from the database was included in the created method in the Agilent Mass Hunter Quantitative Analysis program. If the coefficient of agreement was lower, the abbreviation UN (unknown) was assigned to the *m*/*z* value appearing at a specific retention time. This means that an extracted ion chromatogram was reconstructed for the obtained signals on the mass spectrum, and the presence of a chromatographic peak was confirmed in order to exclude signals constituting noise, and to accurately determine the retention time of the compound and assign *m*/*z* values correctly in the case of partial coelution of compounds. As a result, the most intense signals (threshold 1000 cps) combined with retention time (feature) were selected to track changes in the amounts of unknown compounds. This approach ensured that *m*/*z* signals unrelated to a given compound did not correlate with the other assigned signals.

Based on the analysis of 96 chromatograms, a list of 123 compounds was created. Of these, 67 compounds had specified identities, and 56 were indicated as unknown (see Table 3 for identities proposed for sample F200496_4A). These compounds were characteristic of the synthesis method or the origin of the substrate and fillers added during the drug distribution process [96,97,98,99,100,101]. As each compound is fragmented during electron ionization, each can be represented by multiple signals observed at different *m*/*z* values. It is recommended to observe at least two ions for each compound. Due to potential interferences in complex samples, we decided to monitor at least four ions for each compound. Consequently, a list of 783 potential features (retention time combined with *m*/*z* value) was created to capture a comprehensive chromatographic profile for each sample.

In order to quickly determine the total area of the chromatogram, a peak integration method was established in the Enhanced Data Analysis ChemStation program. Optimal results were obtained for a width of the chromatographic peak of 0.1 min and an initial threshold of 15 in the extracted chromatograms. The obtained peaks’ areas were exported to Excel (Microsoft) software.

During sample preparation for chemometric analysis, various normalization methods were tested, including internal normalization (relative to the total chromatogram area, or to the peak area obtained for the internal standard) and external normalization (relative to the total chromatogram area obtained for the QC sample). The optimal normalization method was identified as internal normalization relative to the total peak area of the entire chromatogram. To facilitate analysis, the area ratios were scaled by multiplying by 10^10^ to obtain integer values. Additionally, main components such as the internal standard, amphetamine, and caffeine were scaled by multiplying their values by 10^5^, in order to reduce their influence on the compared features and enhance the sensitivity of similarity analysis for trace components.

### 2.5. Impurity Profiling

**Figure 7:**
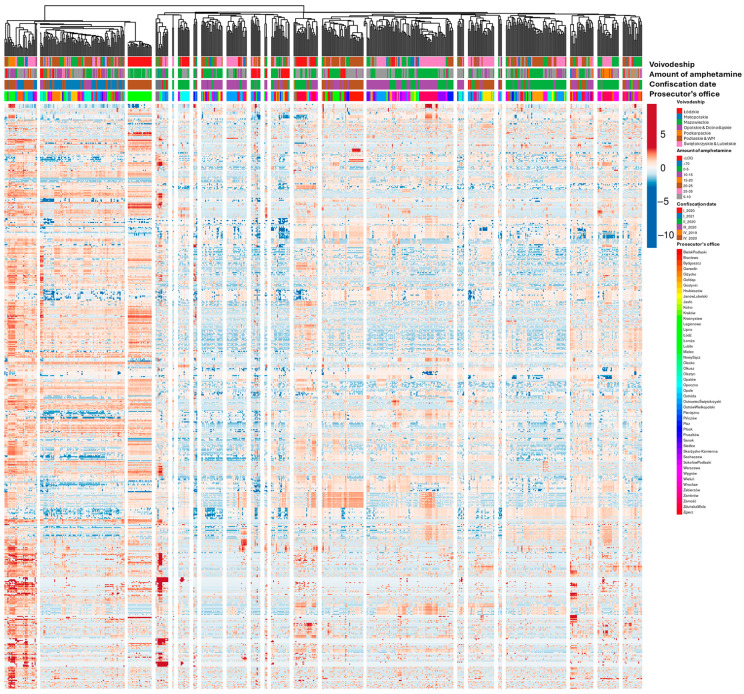
Heat map with 22 indicated clusters based on samples’ composition similarity (r > 0.5), with annotations on top indicating sample clustering. Colors on heat map range from red (highest values) to blue (lowest values), representing Z-score normalized peak areas divided by their standard deviation.

The visualization of the results was performed using the online tool ClustVis [102]. The optimal parameters for analysis were determined to include data transformation using the logarithm function, combined with scaling. The correlation coefficient was identified as the most effective metric for measuring distances between clusters on the heat map. Based on this approach, the heat map revealed 22 distinct clusters of samples characterized by similar impurity profiles (Figure 7 and Figure S1—high resolution). A tentative classification was performed by calculating the correlation coefficients between profiles and visually analyzing the resulting dendrogram. The heat map reveals that the clustering of samples is primarily influenced by the amphetamine content and the date of confiscation. In the first instance, while efforts were made to minimize the impact of the primary components on the compositional description, this inadvertently amplified the influence of fillers and diluents on the sample profiles. Consequently, it became possible to observe the effects of changes in the composition of both major components and impurities.

**Figure 8:**
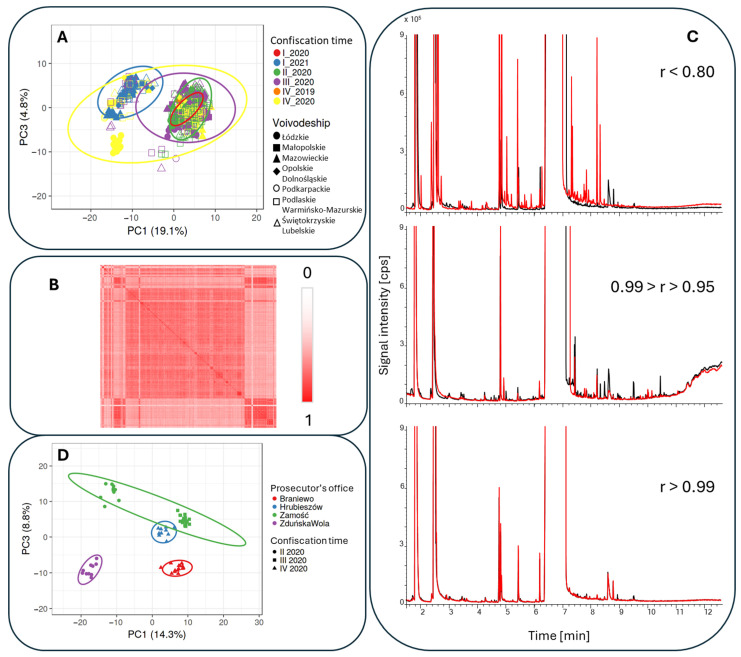
A PCA chart showing that the time of the drug’s presence on the market significantly influenced its composition (**A**). A visual assessment of the similarity matrix (**B**) and the chromatograms, in comparison to the Pearson linear correlation coefficient (**C**). A PCA chart confirming the source affiliation of the samples, indicated by the Pearson linear correlation coefficient (**D**).

In the second case, the disruption of chemical reagent supply chains during the COVID-19 pandemic played a significant role. Notably, samples containing less than 8% amphetamine were predominant throughout 2020, with a marked increase in samples containing less than 2% amphetamine observed in the last quarter of that year (Figure 8A).

It is also evident that in some provinces (voivodeships), seized samples, despite being confiscated at different times, displayed similar compositions and relatively consistent levels of amphetamine and major constituents. This consistency is likely due to minimal dilution within the distribution chain, which was presumably too short to allow significant compositional variation. Consequently, it becomes possible to pinpoint potential centers of origin for the drug.

Our study revealed that the Leuckart reaction remains the most commonly employed method for amphetamine synthesis in Polish illegal laboratories, accounting for 465 out of the 583 analyzed samples. This method is preferred due to its efficiency, enabling the production of amphetamine sulfate within 20–30 h without requiring advanced equipment or specialized expertise, making it accessible to individuals with minimal or no background in chemistry [16].

Following the classification of benzyl methyl ketone (BMK) as a controlled substance in 2009, its availability has significantly decreased. Our findings confirm the adoption of alternative precursors, such as 2-methyl-3-phenyl-2-oxiranecarboxylic acid (BMK methyl glycidate), predominantly imported from China.

The synthesis of amphetamine via the Leuckart reaction generates various byproducts, including 4-methyl-5-phenylpyrimidine, N-formylamphetamine, 4-benzylpyrimidine, N-(β-phenylisopropyl)benzaldimine, and N,N-di-(β-phenylisopropyl)formamide. These compounds are indicative of the synthesis method, reaction conditions, isolation techniques, and purity of the precursors employed. Additionally, caffeine and creatine were identified as the most commonly used diluents in amphetamine samples. Additionally, the method aids in identifying local trends in amphetamine dilution, such as the incorporation of stearic acid in the vicinity of the small city of Garwolin in Mazowieckie province. Leveraging such local trends in amphetamine profiling could prove highly significant. The obtained similarity matrix pattern for the Polish illegal drug market (most of the samples show good similarity, with a correlation coeficient higher than 0.7) indicate that substantial quantities of amphetamine are produced by a similar method. Most amphetamine is probably synthesized by Polish criminal organizations operating in the Netherlands and subsequently smuggled into Poland [103]. The obtained results are in agreement with the investigation results, which show that within Poland, the process is limited to the precipitation of amphetamine salts and the subsequent distribution of the drug.

To visually assess the similarity of chromatograms, characteristic features for each group were determined. To confirm whether the sample data originated from the same source, the Pearson linear correlation coefficient was used as a measure of chromatogram similarity. Based on the value of the correlation coefficient between impurity profiles, the following parameters were established: r > 0.99—samples from the same batch or synthesis run; 0.99 > r > 0.95—samples from the same source; 0.95 > r > 0.80—samples likely from the same source; and 0.80 > r—samples from different sources. The method was validated by comparing chromatograms of 40 randomly selected samples representative of each similarity group (Figure 8B).

The method of comparing amphetamine profiles using the Pearson linear correlation coefficient has proven effective, though it is not without limitations. Challenges in result interpretation can arise when a single peak in the chromatogram has a significantly larger surface area than the others. Such a dominant peak can inflate the correlation coefficient, masking differences in other parts of the chromatogram. To address this, the correlation coefficient was recalculated after data normalization and the removal of influence from the main components. Based on this approach, five clusters were obtained for which the correlation coefficient was higher than 0.8.

Samples from Zamość contained amphetamine at concentrations of approximately 20%, initially adulterated with caffeine via co-precipitation, and later with palmitic acid during distribution. This sequence was inferred from a set of samples from a single laboratory, showing varying purity levels corresponding to different stages of preparation.

Over a three-month period in 2020, the composition of the seized material changed markedly. Despite originating from the same city, the differences in composition indicate that the amphetamine came from different batches. Some samples contained amphetamine concentrations below 5%, with elevated levels of BMK and various methylphenylamine derivatives, which are byproducts of synthesis. These low-amphetamine samples were diluted exclusively with caffeine. In contrast, samples from Braniewo were adulterated with both caffeine and benzoic acid. Meanwhile, samples from Hrubieszów and Zduńska Wola were synthesized using the Leuckart method, but displayed differing intensity ratios of specific synthesis markers, highlighting variations in production techniques.

To evaluate the potential of Principal Component Analysis (PCA) in verifying whether a sample analyzed via the established GC-MS method belonged to its assigned source, we used data from samples associated with the five potential sources indicated by the Pearson linear correlation coefficient. The PCA results, shown in Figure 8D, demonstrate that the clusters formed align with the location and timing of the drug seizures. This consistency indicates that PCA can effectively support source identification by grouping samples based on their compositional similarities.

It should be noted, however, that as sample dilution increases, the compositional similarity diminishes, reducing the likelihood of accurately identifying the source. Another critical factor influencing composition is the availability of chemical reagents. Consequently, the seizure date of material associated with the short operational window of an illegal laboratory becomes a key factor in linking the laboratory to its distribution network.

## 3. Materials and Methods

### 3.1. Chemicals and Reagents

An internal standard solution (ISTD) of N,N-dimethylbenzylamine (HPLC-grade, Sigma-Aldrich, Saint Louis, MO, USA) with a concentration of 2 g/L was prepared by mixing 2 g of liquid, quantita-tively transferred to a 1 L volumetric flask, with a mixture of methanol (pure for analysis, POCH, Gliwice, Poland) and water (LC-MS purity, VWR, Radnor, PA, USA) in a ratio of 8:2 (*v*/*v*). The solution was stored in a refrigerator at 4 °C. Standard solutions for the amphetamine calibration curve, with concentrations of 5–200 µg/mL, were prepared by diluting a certified amphetamine standard with a concentration of 1 mg/mL in methanol (LC-MS grade, Sigma-Aldrich, Saint Louis, MO, USA), to which a constant volume of a twofold diluted ISTD solution was added (25 µg/mL in each solution). The solutions were stored at −20 °C.

### 3.2. Registration of Evidence and Collection of Analytical Samples

From 2020 to 2021, evidence from selected street seizures conducted by law enforcement agencies across Poland was submitted to the MASDIAG laboratory for analysis (See Figure S1 for an example of sample received). Upon receipt, critical information related to each piece of evidence was logged in the laboratory database. This included a unique identification number, details of the law enforcement unit handling the criminal case, and the date and location of the seizure.

The database was subsequently updated with specific sample characteristics such as gross weight, net weight, form of the seized powder, drug concentration, diluents, number of intoxicating doses, intoxicating mass, commercial portions, market value, and any additional pertinent details. Photographic documentation of each submitted sample was also created for reference.

For sample analysis, when the total number of samples was ten or fewer, all samples were analyzed. However, for quantities exceeding ten, the number of samples to be analyzed was determined using the square root method for calculating sample size. The sampling plan, including the number of samples analyzed based on the total sample size, is outlined in Table 4.

### 3.3. Sample Preparation Procedure

Evidence in the form of powder was initially weighed and homogenized using a mortar. A representative sample (0.20–0.30 g) was accurately measured on analytical balances (AS 310.R2, AS 120 R2 PLUS, RADWAG, Warsaw, Poland) and transferred into a 15 mL Falcon tube. The samples were extracted with 10 mL of an 8:2 MeOH:H_2_O mixture containing an internal standard (ISTD) at a concentration of 1 g/L. The extraction process was facilitated by shaking at 70 RPM for 30 min using an Elmi Rotator Intelli-Mixer RM-2L. After extraction, the samples were centrifuged at 5800 RPM for 5 min at room temperature with an MPW-54 centrifuge (MPW MED INSTRUMENTS, Warsaw, Poland).

A 25 µL aliquot of the supernatant was transferred into a glass vial with a septum-sealed cap for gas chromatography. Subsequently, 975 µL of MeOH was added to the vial, and the sample was analyzed by GC-MS. If amphetamine was detected in the sample, 1 mL of the supernatant was subjected to further analysis using GC-MS.

### 3.4. Instrumentation

Instrumental analysis was performed using an Agilent HP 6890 Plus gas chromato-graph (Agilent Technologies, Santa Clara, CA, USA), equipped with a GC PAL CTC Analytics autosampler (CTC Analytics, Zwingen, Switzerland) and an Agilent HP 5973 mass spectrometer detector (Agilent Technologies, Santa Clara, CA, USA). The capillary column HP-1 17 m × 0.20 mm × 0.11 µm (Agilent Technologies, Santa Clara, CA, USA) was applied to separate compounds in the extracted samples.

### 3.5. Calibration of Method for Determination of Amphetamine

Amphetamine standard solutions with concentrations ranging from 5 to 200 µg/mL were prepared by diluting a 1 mg/mL amphetamine stock solution in methanol, with a constant volume of a twofold diluted solution of ISTD. Calibration curves were generated by plotting the ratio of the amphetamine peak area to the internal standard peak area (P_AMF_/P_ISTD_) for five different amphetamine concentrations. Curves were recalibrated approximately once per month. During the study, the injector filter of the gas chromatograph had to be replaced, which resulted in reduced method sensitivity. Consequently, the calibration range was adjusted to 10–200 µg/mL. For each amphetamine concentration in standard solutions, three replicates (n = 3, technical repetitions) were analyzed. At concentrations of 5, 10, and 200 μg/mL, the coefficient of variation often exceeded 5%, whereas for other concentrations, it typically remained below 2% (Table 5).

The determined limit of detection (LOD) for amphetamine was 3.87 μg/mL, and the limit of quantification (LOQ) was 11.60 μg/mL. These values were calculated based on five different amphetamine concentrations (*p* = 5), each analyzed in quintuplicate (n = 5). The LOD was calculated using the formula LOD = 3.3⋅a/Sb, where a represents the slope of the calibration curve, and Sb is the standard error of the intercept.

The detection and quantification limits achieved with the GC-MS method are comparable to those obtained with GC-FID [34,35] and HPLC-UV [68] methods. However, using less-selective detectors required the analysis time to be extended (up to 30–40 min) to improve selectivity, including the capability to separate and determine enantiomers via chiral liquid chromatography. Sensitivity and selectivity can be further enhanced by sample concentration techniques, such as liquid–liquid extraction (LLE) or solid-phase extraction (SPE) [76,81].

Significant improvements in sensitivity, up to a thousandfold, can also be achieved using a tandem mass spectrometer in MRM (Multiple Reaction Monitoring) mode, which selectively monitors predefined reactions without increasing analysis time [76,79,81]. While this approach greatly enhances sensitivity, it is limited to tracking known compounds with established fragmentation pathways, resulting in a trade-off, where information about unknown compounds is lost.

### 3.6. Precision of Quantitative Method

Precision was evaluated by calculating the standard deviation (SD) and relative standard deviation (RSD) of the mean analyte concentration. To assess the repeatability of determinations, five independent samples of fine powder evidence were analyzed using GC-MS. This approach allowed for consideration of the influence of the sample’s primary components on the consistency of the results.

The high standard deviation (Table 6) could be attributed to the lack of homogeneity in the materials under investigation. Using an internal standard to calibrate amphetamine concentration eliminates issues associated with sample preparation. A substantial increase in sample weight would be required to avoid the issue of homogeneity. The level of active substances in the seized material can fluctuate greatly, impacting the quantity of the sample that needs to be weighed. Conducting multiple extractions, dilutions, and analyses for varying quantities of powder greatly elevates the cost and increases the time of the analysis, and complicates efforts to precisely regulate the dosage of the drug throughout the standardized analytical procedure.

The accuracy and precision of the method depend on the efficiency of sample transport from the syringe through the GC dispenser to head of the chromatographic column, as well as the sample’s recovery from the stationary phase. Methanol post-sample analysis was carried out for each illicit sample to detect “sample carry over” effects. Each chromatogram for methanol was scrutinized for peaks associated with amphetamine, caffeine, and other commonly found primary compounds in substances of misuse. In each instance, the chromatogram obtained for methanol showed no peaks with amplitudes exceeding three times the baseline of the chromatogram.

### 3.7. Long-Term Precision and Quality Assurance for GC-MS

**Figure 9:**
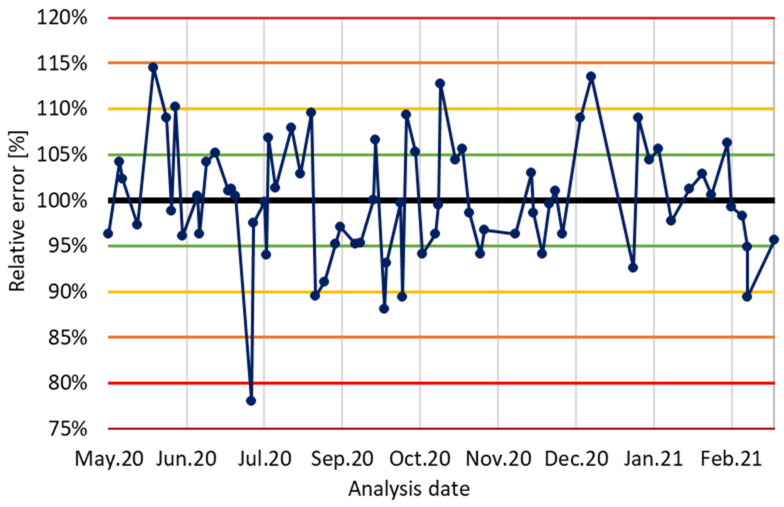
Shewhart’s chart, illustrating variations in established amphetamine concentrations for QC samples throughout study, in relation to nominal value.

The repeatability of the method was evaluated by analyzing a quality control (QC) sample containing an internal standard (ISTD) and amphetamine, alongside a randomly selected test sample. It was defined as the accuracy of the determined concentration in a control sample with a known amphetamine concentration of 50 μg/mL (the nominal value). The outcome was displayed as relative deviation and plotted on a Shewhart control chart (Figure 9).

The acceptable range for relative deviation (RD) was set between 80 and 120%. Determinations were conducted if the variation in amphetamine concentration from one day to the next was not excessively large, or if the measurement points did not exhibit a systematic increase or decrease. In cases where significant spikes or falls were observed, or when systematic trends in the data were detected, the instrument was cleaned, and measurements were postponed until proper functioning was restored.

The developed method, though less selective than liquid chromatography-based techniques, is sufficient for its intended purpose and offers good intermediate precision. Long-term studies necessitated regular maintenance of the instrument and monitoring of its cleanliness to ensure consistent reproducibility. Systematic cleaning of the interface zone and frequent replacement of the inlet liner (weekly instead of monthly) were essential, due to the high injector temperature, the number of samples analyzed (usually 30–50 daily), and the presence of polar and less-volatile compounds in the samples.

Additional unscheduled maintenance of the injector (inlet liner replacing and interface cleaning) was occasionally required to address the accumulation of non-volatile compounds, such as amine salts or powdered sugar, used to dilute the psychoactive substances. For instance, interventions were needed on 20 of July 2020 (Figure 9) to clean the interface, after quality control (QC) revealed amphetamine concentrations outside of acceptable ranges. Moreover, when QC deviations or trends were observed over two or three consecutive points (for example on 21 February 2021), immediate cleaning of the interface section was performed to restore accuracy (Table 7).

### 3.8. Data Analysis

Quantitative analysis was performed using the Agilent Mass Hunter Quantitative Analysis software (B.05.00 SP02/5.0.291.4, Agilent Technologies, Santa Clara, CA, USA).

Data normalization was carried out using Excel (Microsoft) software. Cluster analysis, heat maps, and principal component analysis were preformed using the ClustVis application [102].

## 4. Conclusions

This study highlights the effectiveness of GC-MS, combined with chemometric methods, for the forensic profiling of amphetamines. By identifying synthesis markers and differentiating samples based on their chemical profiles, this approach offers valuable insights for law enforcement and public health strategies. The profiling methodology provides critical data on trends in precursor usage, regional distribution patterns, and adulteration practices, serving as a powerful tool for understanding the dynamics of the illicit amphetamine market. Scaling up the implementation of these methods could significantly enhance efforts to trace production networks and disrupt their operations effectively.

The analytical method developed in this research demonstrated high intermediate precision, even when analyzing samples containing compounds with significantly different volatilities. This required a stringent approach to maintaining the cleanliness of the injector and interface components, to ensure the necessary sensitivity. The method successfully achieved its objectives, enabling the separation and detection of a wide array of compounds associated with the synthesis process, as well as those introduced during drug distribution. The impurity profiling technique proved instrumental in determining precursors, identifying the synthesis method, and pinpointing the main diluents. Furthermore, this profiling capability facilitated the classification of samples into groups based on their origin.

The origins of the samples were verified using the Pearson linear correlation coefficient and visual evaluation of chromatograms. It was also demonstrated that the source of the drug could be confirmed by analyzing the distribution of principal components from selected data sets. A classification system based on Pearson coefficient values was effectively employed to group samples according to their sources.

## Figures and Tables

**Table 1:** Optimal parameters of GC-MS analytical method.

Carrier gas type and flow	helium, 1 mL/min
Volume of sample injected	2 μL
Injector type	1:10 split mode
Injector temperature	250 °C
Column temperature program	0.0–0.2 min–75 °C (0 °C/min)
	0.2–0.5 min–90 °C (10 °C/min)
	0.5–1.0 min–325 °C (25 °C/min)
Transfer line temperature	310 °C
Ion source temperature	230 °C
Electron bombardment energy	70 eV
Mass scanning range	50–550 amu

**Table 2:** Summary of information related to analyzed samples (n = 583).

The average concentration of amphetamine in the analyzed samples	7.6%
The median concentration of amphetamine in the analyzed samples	6.2%
Samples with a dominant amount of amphetamine	84.4%
The total mass of the analyzed evidence material containing amphetamine [g]	8113
The estimated total market value of the analyzed evidence [EUR]	81,132

**Table 3:** Proposed identities of compounds detected in sample F200496_4A—chromatogram presented in Figure 7. Retention indices from National Institute of Standards and Technology (NIST) spectra library (version 17). Scores were calculated for mass spectra after noise substraction. Proposed identities were further verified against literature reports on amphetamine-related contaminants [96,97,98,99,100,101].

Peak Number	t_R_ [min]	Retention Indices	*m*/*z* for Four Most Intense Signals in Mass Spectrum	Proposed Identity	Score [%]
1	1.8	1042	135, 91, 65, 58	N,N-dimethylbenzylamine, ISTD	90
2	2.4	1128	134, 92, 91, 65	Benzyl methyl ketone, BMK	59
3	2.5	1171	120, 91, 65, 51	Amphetamine, AMF	75
4	4.7	1468	170, 169, 115, 102	4-methyl-5-phenyl pyrimidine	60
5	4.8	1415	118, 91, 72, 51	N-formyl amphetamine	62
6	4.8	1454	170, 169, 91, 72	4-benzylpyrimidine	68
7	5.4	1663	91, 71, 56, 42	4-methylphenmetrazine	58
8	6.2	1896	132, 105, 91, 77	N-(-phenylisopropyl)benzaldimine	65
9	7.1	1795	194, 109, 82, 67	Caffeine	99
10	8.6	-	280, 221, 208, 194	Unknown, UN	-
11	8.8	-	281, 235, 194, 150	Unknown, UN	-

**Table 4:** Sample selection algorithm.

Total Number of Packages	Number of Packages Taken for Testing
n < 10	n
10 < n < 100	10
n > 100	√n

**Table 5:** The determined standard deviation (STD) and coefficient of variation (CV) for individual points of the standard curves.

Time	Curve’s Equation	R^2^	C_AMF_ [μg/mL]	STD [μg/mL]	CV [%]
11 May 2020	y = 0.0115x + 0.0253	0.9994	5	0.0026	3.3
			10	0.0038	2.9
			50	0.0108	1.8
			100	0.0268	2.2
			200	0.0281	1.2
1 June 2020	y = 0.0119x − 0.0214	0.9950	5	0.0007	1.6
			10	0.0023	2.7
			50	0.0005	0.1
			100	0.0118	1.0
			200	0.0311	1.2
7 July 2020	y = 0.0166x + 0.0022	0.9954	5	0.0005	0.8
			10	0.0023	1.7
			50	0.0031	0.4
			100	0.0087	0.5
			200	0.1404	4.3
30 July 2020	y = 0.0115x + 0.0154	0.9983	5	0.0110	15
			10	0.0007	0.6
			50	0.0191	3.1
			100	0.0687	5.9
			200	0.0092	0.4
15 September 2020	y = 0.0132x − 0.0379	0.9967	10	0.0025	2.7
			25	0.0072	2.5
			50	0.0073	1.2
			100	0.0212	1.6
			200	0.1385	5.3
1 October 2020	y = 0.0123x + 0.0330	0.9960	10	0.0034	3.9
			25	0.0011	0.4
			50	0.0037	0.6
			100	0.0364	3.0
			200	0.0106	0.5
11 January 2021	y = 0.0140x + 0.0732	0.9986	10	0.0013	0.7
			25	0.0381	8.5
			50	0.0270	3.3
			100	0.0017	0.1
			200	0.0004	0.1
3 March 2021	y = 0.0103x − 0.0436	0.9940	10	0.0045	7.3
			25	0.0053	2.6
			50	0.0356	7.9
			100	0.0302	3.0
			200	0.0958	4.5
23 March 2021	y = 0.0124x − 0.0707	0.9941	10	0.0042	7.2
			25	0.0086	3.9
			50	0.0223	4.2
			100	0.0418	3.39
			200	0.0886	3.55

**Table 6:** Preparative (and technical) precision of GC-MS method.

Weight [g]	Amphetamine Concentration [%]	Mean Amphetamine Concentration [%]	SD	RSD [%]
0.2025	5.94	6.56	0.86 (0.50)	13.09 (6.60)
0.2044	6.88			
0.2017	6.46			
0.2017	6.88			
0.2019	6.64			

**Table 7:** Maintenance tasks performed on GC-MS instrument.

Task	Frequency
Replacing the septa	Every week
Replacing the inlet liner	Every week
Cleaning of interface components	Every three months
Replacing the filament	Every three months
Replacing the chromatographic column	Every year After 3000 samples
Checking the sensitivity of the instrument by means of a QC sample	Before each sequence on the day of analysis

## Data Availability

Data on the analytical results, without data obtained by the Police during investigation, can be made available upon request.

## Supplementary Materials

The following supporting information can be downloaded at https://www.mdpi.com/article/10.3390/molecules30030579/s1: Figure S1: Picture of confiscated amphetamine in form of powder.
