# Supplementary figures and images for: Comprehensive Profiling of Illicit Amphetamines Seized in Poland: Insights from Gas Chromatography–Mass Spectrometry and Chemometric Analysis
Source: Molecules. 2025 Jan 27;30(3):579. doi: 10.3390/molecules30030579 (PMC11819816; doi:10.3390/molecules30030579)

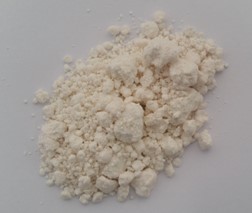

Supplement: Supplementary file 1 [file molecules-30-00579-s001.zip › molecules-3431914-supplementary.jpg]
